# Testing a digital system that ranks the risk of unplanned intensive care unit admission in all ward patients: protocol for a prospective observational cohort study

**DOI:** 10.1136/bmjopen-2019-032429

**Published:** 2019-09-11

**Authors:** James Malycha, Oliver C Redfern, Guy Ludbrook, Duncan Young, Peter J Watkinson

**Affiliations:** 1 Nuffield Department of Clinical Neurosciences, University of Oxford, Oxford, UK; 2 Kadoorie Centre for Critical Care Research and Education, Oxford University Hospitals NHS Foundation Trust, Oxford, Oxfordshire, UK; 3 Faculty of Health and Medical Sciences, University of Adelaide, Adelaide, South Australia, Australia

**Keywords:** clinical deterioration, intensive care unit, critical care unit, predictive score, electronic patient record, qualitative medical note review

## Abstract

**Introduction:**

Traditional early warning scores (EWSs) use vital sign derangements to detect clinical deterioration in patients treated on hospital wards. Combining vital signs with demographics and laboratory results improves EWS performance. We have developed the Hospital Alerting Via Electronic Noticeboard (HAVEN) system. HAVEN uses vital signs, as well as demographic, comorbidity and laboratory data from the electronic patient record, to quantify and rank the risk of unplanned admission to an intensive care unit (ICU) within 24 hours for all ward patients. The primary aim of this study is to find additional variables, potentially missed during development, which may improve HAVEN performance. These variables will be sought in the medical record of patients misclassified by the HAVEN risk score during testing.

**Methods:**

This will be a prospective, observational, cohort study conducted at the John Radcliffe Hospital, part of the Oxford University Hospitals NHS Foundation Trust in the UK. Each day during the study periods, we will document all highly ranked patients (ie, those with the highest risk for unplanned ICU admission) identified by the HAVEN system. After 48 hours, we will review the progress of the identified patients. Patients who were subsequently *admitted* to the ICU will be removed from the study (as they will have been correctly classified by HAVEN). Highly ranked patients *not admitted* to ICU will undergo a structured medical notes review. Additionally, at the end of the study periods, all patients who had an unplanned ICU admission but whom HAVEN *failed to rank highly* will have a structured medical notes review. The review will identify candidate variables, likely associated with unplanned ICU admission, not included in the HAVEN risk score.

**Ethics and dissemination:**

Approval has been granted for gathering the data used in this study from the South Central Oxford C Research Ethics Committee (16/SC/0264, 13 June 2016) and the Confidentiality Advisory Group (16/CAG/0066).

**Discussion:**

Our study will use a clinical expert conducting a structured medical notes review to identify variables, associated with unplanned ICU admission, not included in the development of the HAVEN risk score. These variables will then be added to the risk score and evaluated for potential performance gain. To the best of our knowledge, this is the first study of this type. We anticipate that documenting the HAVEN development methods will assist other research groups developing similar technology.

**Trial registration number:**

ISRCTN12518261

**Strengths and limitations of this study**

- The study methodology is in accordance with the STrengthening the Reporting of OBservational studies in Epidemiology guidelines.
- We describe a method that combines risk score testing with a structured medical notes review conducted by a clinical expert for the iterative improvement of a digital system that quantifies risk for unplanned intensive care unit admission in all ward patients.
- To the best of our knowledge, this is the first study of this type.

## Background

### Introduction

Early warning score (EWS) systems, such as the National Early Warning Score, combine abnormalities in patient vital signs into an aggregate score.^1^ This score triggers a clinical response when a threshold is exceeded. Despite wide-scale adoption of EWS systems, significant clinical patient deterioration on hospital wards still occurs.^1 2^ Additionally, high numbers of false alerts lead to alert ‘fatigue’ and inefficient use of response teams.^3^ Adding additional clinical information to such systems, such as laboratory results and comorbidities, improves specificity.^4–12^ However, identifying and adding new variables requires a systematic approach to avoid needless complexity.^13^

We have developed a system to predict the risk of unplanned intensive care unit (ICU) admission (within 24 hours) for patients on general medical and surgical wards. It is called the Hospital Alerting Via Electronic Noticeboard (HAVEN).^14^ To identify potential variables for inclusion in HAVEN, we used a modified Delphi process and a systematic literature review.^15^ Those identified variables that were available within the electronic patient record (EPR) were extracted from data sets comprising all patients admitted to two National Health Service (NHS) trusts (a trust is a legal entity that provides goods and services for the purposes of the provision of hospital, community and/or other aspects of patient care).^12^ We then used a machine learning method^16^ to select the optimal combination of variables for the HAVEN risk score. In contrast to EWS systems, HAVEN was not designed to produce alerts. Instead, HAVEN provides a list of patients in the hospital, ranked from most to least at risk of requiring ICU admission. The intent is that HAVEN will improve patient safety by informing the use of clinical response teams.

### Aims and objectives

The primary aim of this study is to discover additional candidate variables, not recognised during the data-driven derivation process, that would improve the performance of the HAVEN risk score. We will review the medical records of *misclassified* patients, that is, patients ranked highly by HAVEN but who were not admitted to the ICU; or patients who were never ranked highly by HAVEN but had an unplanned ICU admission.

### The HAVEN risk score

The HAVEN risk score is calculated using both *static* and *dynamic* variables extracted in real time from the EPR.

Static variables refer to patient-level data available at admission: age, gender, comorbidities (classified according to the Elixhauser comorbidity index^17^) and Hospital Frailty Risk Score.^18^ As diagnostic coding in the UK occurs after a patient has been discharged, the comorbidity index and frailty scores are calculated using a patient’s admissions over the previous 2 years. Score performance in patients with no previous admissions (and potentially undocumented comorbidities) will be evaluated separately.

Dynamic variables refer to measurements taken repeatedly during hospital admission, that is, laboratory results and vital signs. The HAVEN risk score is currently updated according to the most recent measurements of: albumin, bilirubin, C reactive protein, haemoglobin, platelets, white cell count, potassium, sodium, urea, creatinine, heart rate, systolic blood pressure, respiratory rate, body temperature, a neurological status assessment using either the Alert-Verbal-Painful-Unresponsive scale or the Glasgow Coma Scale, peripheral oxygen saturation from pulse oximetry (SpO_2_) and the estimated fraction of inspired oxygen.^19^ A patient’s HAVEN score is re-calculated each time a new dynamic variable is received by the system and the score is further adjusted for the time since hospital admission.

## Methods

The study will be reported according to the STrengthening the Reporting of OBservational studies in Epidemiology guidelines.^20^

### Design and setting

This is a prospective, observational, cohort study conducted at the John Radcliffe Hospital, part of the Oxford University Hospitals NHS Foundation Trust in the UK. The John Radcliffe Hospital is a tertiary hospital with over 800 beds and serves a population of over 650 000 people, who are generally more affluent and with higher life expectancy than the national average.^21^

## Data collection

Data collection will occur during 4, full, non-consecutive weeks in 2019. The notes review will be undertaken by a senior critical care physician. Patients who are discharged or die during the study period will have these details recorded. They will remain in the analysis data set.

### Participants

#### Eligibility criteria

Emergency and elective adult patients (16 years or over) admitted to medical, surgical, observational or short-stay wards will be eligible for inclusion. We will exclude patients for whom a score cannot be generated (ie, those with no recorded vital sign or laboratory measurements).

#### Sample size

We will sample two subgroups of patients:

1. False High Rank (FHR).
2. False Low Rank (FLR).

The FHR group will consist of patients ranked highly by HAVEN but who were not admitted to the ICU. To identify this group, we will record the five highest-ranked patients on the HAVEN system at 09:00 each morning of the study. After 48 hours, we will remove any patients who were subsequently admitted to the ICU. The remaining patients’ records will be reviewed.

The FLR group will be identified at the end of the study and consist of all patients who had an unplanned ICU admission during the study period and were not present in any of the daily high-ranking groups. These patients’ records will also undergo a medical notes review.

The study will run for 4 non-consecutive weeks with expected recruitment of between 130 and 150 patients.

### Structured medical notes review

We will carry out a structured review of patient medical notes (electronic and paper-based) for the two sample groups described in section Sample size. From these, we will construct a medical summary, looking specifically at patient-centred and system-based variables associated with decisions around ICU admission. We will use a modified version of the Hogan *e* *t* *al* qualitative note review techniques.^22^ We will then conduct a thematic analysis of the extracted data.^23^ It is expected that from within the themes the additional variables will be identified. Along with the *as yet unknown* variables, the following data will be extracted:

1. Primary diagnosis.
2. Comorbidities and medical history (where not available from previous admissions).
3. Any treatment limitations put in place and the reasons for these, including ‘Do not attempt resuscitation’ documents.
4. Current medication.
5. Radiological imaging.
6. Point-of-care blood gas analysis.
7. Clinical Frailty Score.^24^

### Qualitative methods

Qualitative data (eg, information in free text) will be analysed thematically, using methods of constant comparison.^25^ A coding framework will be constructed to assist in understanding of the data. We will use NVivo software (QSR International, www.qsrinternational.com) to support the qualitative analysis process.

### Patient safety and public involvement

As an observational study of patient records with no intervention, adverse events related to research interventions are not possible. In the event that inadequate care is identified during the structured medical note review, local NHS trust protocols will be followed. Reviewers will act in accordance with the General Medical Councils Good Medical Practice Guidelines (2013). This action includes acting immediately if a patient is not receiving basic care to meet their needs. If patients are at risk because of inadequate premises, equipment or other resources, and policies or systems, we will correct the matter if possible and raise our concerns in line with workplace policy. All measures will be documented as per local policies. The HAVEN project has had two lay members on the management committee throughout. They have been involved in regular discussions regarding the aims and remit of the HAVEN project.

## Discussion

### Main findings

This study will use structured medical notes review on ward patients misclassified by HAVEN to identify variables that may enhance performance. Any identified variables will be systematically introduced into our score development pipeline to evaluate whether they improve score performance.

### Strengths and limitations of the study

This study is part of a project-wide process to document the development of the HAVEN system such that it is thorough, transparent, repeatable, reportable and the methodology could be useful for other groups developing similar technology.

Unplanned ICU admission is an outcome measure subject to bias, such as the decision-making of individual physicians, local practice guidelines and bed availability.^26 27^ This study is limited to one hospital and the results may not be generalisable to other hospitals. Variables identified from the thematic analysis may not be available in the EPR and therefore unable to improve the performance of the HAVEN risk score. Likewise, patients with no previous admissions to the John Radcliffe Hospital will have no available comorbidity data, potentially limiting the performance of the risk score in these patients. To assess the impact of these missing data, we will undertake subgroups analyses in those patients with/without prior admissions.

While a significant proportion of ICU admissions are referred directly from the emergency department (ED), the HAVEN system was designed specifically for ward patients needing the attention of the critical care team. By excluding these ED referrals, we are reducing the number of eligible patients for this study.

### Implications

To the best of our knowledge, this is the first protocol to describe a study of this type. We hope this protocol will assist future development of similar systems.

## Supplementary Material

Reviewer comments

Author's manuscript

## References

[1] Royal College of Physicians (London) National early warning score (news) 2: standardising the assessment of acute-illness severity in the NHS, 2017.

[2] Care Quality Commission Opening the door to change NHS safety culture and the need for transformation, 2018.

[3] Curry JP, Jungquist CR A critical assessment of monitoring practices, patient deterioration, and alarm fatigue on inpatient wards: a review. Patient Saf Surg 2014;8:29 10.1186/1754-9493-8-29 25093041 PMC4109792

[4] Churpek MM, Yuen TC, Park SY, et al Using electronic health record data to develop and validate a prediction model for adverse outcomes in the Wards*. Crit Care Med 2014;42:841–8. 10.1097/CCM.0000000000000038 24247472 PMC3959228

[5] Kang MA, Churpek MM, Zadravecz FJ, et al Real-Time risk prediction on the wards: a feasibility study. Crit Care Med 2016;44:1468–73. 10.1097/CCM.0000000000001716 27075140 PMC4949091

[6] Alvarez CA, Clark CA, Zhang S, et al Predicting out of intensive care unit cardiopulmonary arrest or death using electronic medical record data. BMC Med Inform Decis Mak 2013;13:28 10.1186/1472-6947-13-28 23442316 PMC3599266

[7] Bailey TC, Chen Y, Mao Y, et al A trial of a real-time alert for clinical deterioration in patients hospitalized on general medical wards. J Hosp Med 2013;8:236–42. 10.1002/jhm.2009 23440923

[8] Escobar GJ, LaGuardia JC, Turk BJ, et al Early detection of impending physiologic deterioration among patients who are not in intensive care: development of predictive models using data from an automated electronic medical record. J Hosp Med 2012;7:388–95. 10.1002/jhm.1929 22447632

[9] Hackmann G, Chen M, Chipara O, et al Toward a two-tier clinical warning system for hospitalized patients. AMIA. Annu Symp proceedings AMIA Symp 2011;2011:511–9. PMC3243239 22195105

[10] Tam V, Frost SA, Hillman KM, et al Using administrative data to develop a nomogram for individualising risk of unplanned admission to intensive care. Resuscitation 2008;79:241–8. 10.1016/j.resuscitation.2008.06.023 18691801

[11] Kipnis P, Turk BJ, Wulf DA, et al Development and validation of an electronic medical record-based alert score for detection of inpatient deterioration outside the ICU. J Biomed Inform 2016;64:10–19. 10.1016/j.jbi.2016.09.013 27658885 PMC5510648

[12] Redfern OC, Pimentel MAF, Prytherch D, et al Predicting in-hospital mortality and unanticipated admissions to the intensive care unit using routinely collected blood tests and vital signs: development and validation of a multivariable model. Resuscitation 2018;133:75–81. 10.1016/j.resuscitation.2018.09.021 30253229 PMC6562198

[13] Collins GS, Reitsma JB, Altman DG, et al Transparent reporting of a multivariable prediction model for individual prognosis or diagnosis (TRIPOD): the TRIPOD statement. Eur Urol 2015;67:1142–51. 10.1016/j.eururo.2014.11.025 25572824

[14] Watkinson P, Young JD, Prytherch D, et al Hospital alerting via electronic Noticeboard (Haven) study protocol, 2016 Available: https://ora.ox.ac.uk/objects/uuid:322561c7-866e-4c

[15] Malycha J, Bonnici T, Clifton DA, et al Patient centred variables with univariate associations with unplanned ICU admission: a systematic review. BMC Med Inform Decis Mak 2019;19:98 10.1186/s12911-019-0820-1 31092256 PMC6521409

[16] Chen T, He T, Benesty M, et al Extreme Gradient Boosting Package ‘xgboost’. 54, 2018.

[17] Quan H, Sundararajan V, Halfon P, et al Coding algorithms for defining comorbidities in ICD-9-CM and ICD-10 administrative data. Med Care 2005;43:1130–9. 10.1097/01.mlr.0000182534.19832.83 16224307

[18] Gilbert T, Neuburger J, Kraindler J, et al Development and validation of a hospital frailty risk score focusing on older people in acute care settings using electronic Hospital records: an observational study. The Lancet 2018;391:1775–82. 10.1016/S0140-6736(18)30668-8 PMC5946808 29706364

[19] Malycha J, Farajidavar N, Pimentel MAF, et al The effect of fractional inspired oxygen concentration on early warning score performance: a database analysis. Resuscitation 2019;139:192–9. 10.1016/j.resuscitation.2019.04.002 31005587 PMC6547016

[20] von Elm E, Altman DG, Egger M, et al The strengthening the reporting of observational studies in epidemiology (STROBE) statement: guidelines for reporting observational studies. J Clin Epidemiol 2008;61:344–9. 10.1016/j.jclinepi.2007.11.008 18313558

[21] Public Health England Oxford health profile, 2017.

[22] Hogan H, Healey F, Neale G, et al Preventable deaths due to problems in care in English acute hospitals: a retrospective case record review study. BMJ Qual Saf 2012;21:737–45. 10.1136/bmjqs-2011-001159 PMC3436096 22927487

[23] Braun V, Clarke V Using thematic analysis in psychology. Qual Res Psychol 2006;3:77–101. 10.1191/1478088706qp063oa

[24] LS K Frailty in the elderly: a concept analysis. J Nurs 2013;60:105–10. 10.6224/JN.60.1.105 23386532

[25] Huo X, Liu C, Bai X, et al Rsc advances aqueous extract of Cordyceps sinensis potentiates the antitumor E FF ECT of DDP and attenuates. RSC Adv 2017;7:37743–54.

[26] Robert R, Coudroy R, Ragot S, et al Influence of ICU-bed availability on ICU admission decisions. Ann Intensive Care 2015;5:1–7. 10.1186/s13613-015-0099-z 26714805 PMC4695477

[27] Robert R, Reignier J, Tournoux-Facon C, et al Refusal of intensive care unit admission due to a full unit: impact on mortality. Am J Respir Crit Care Med 2012;185:1081–7. 10.1164/rccm.201104-0729OC 22345582

