# Supplementary material for: Testing a digital system that ranks the risk of unplanned intensive care unit admission in all ward patients: protocol for a prospective observational cohort study
Source: BMJ Open. 2019 Sep 11;9(9):e032429. doi: 10.1136/bmjopen-2019-032429 (PMC6747664; doi:10.1136/bmjopen-2019-032429)
Supplement: Author's manuscript [file bmjopen-2019-032429.draft_revisions.pdf]

BMJ Open

BMJ Open is committed to open peer review. As part of this commitment we make the peer review history of every article we publish publicly available.

When an article is published we post the peer reviewers' comments and the authors' responses online. We also post the versions of the paper that were used during peer review. These are the versions that the peer review comments apply to.

The versions of the paper that follow are the versions that were submitted during the peer review process. They are not the versions of record or the final published versions. They should not be cited or distributed as the published version of this manuscript.

BMJ Open is an open access journal and the full, final, typeset and author-corrected version of record of the manuscript is available on our site with no access controls, subscription charges or pay-per-view fees (http://bmjopen.bmj.com).

If you have any questions on BMJ Open's open peer review process please email info.bmjopen@bmj.com

BMJ Open

Testing a digital system that ranks the risk of unplanned Intensive Care Unit admission in all ward patients: protocol for a prospective observational cohort study

Journal:	*BMJ Open*
Manuscript ID	bmjopen-2019-032429
Article Type:	Protocol
Date Submitted by the Author:	18-Jun-2019
Complete List of Authors:	Malycha, James; University of Oxford, Nuffield Department of Clinical Neurosciences;<br>Redfern, Oliver; Oxford University, Nuffield Department of Clinical Neurosciences;<br>Ludbrook, Guy; University of Adelaide,<br>Young, Duncan; Oxford University, Nuffield Department of Clinical Neurosciences<br>Watkinson, Peter; Oxford University Hospitals NHS Trust, Kadoorie Centre for Critical Care research and Education
Keywords:	Clinical deterioration, INTENSIVE & CRITICAL CARE, Predictive score, Electronic Patient Record

SCHOLARONE™
Manuscripts

TITLE PAGE

Testing a digital system that ranks the risk of unplanned Intensive Care Unit admission in all ward patients: protocol for a prospective observational cohort study

AUTHORS
Dr James Malycha (corresponding author) MBBS FCICM
Clinical Research Fellow
Kadoorie Centre for Critical Care Research and Education
Nuffield Department of Clinical Neurosciences
University of Oxford
Level 3, John Radcliffe Hospital,
Headley Way,
Oxford,
OX3 9DU

Postal address: Kadoorie Centre for Critical Care Research and Education
Nuffield Department of Clinical Neurosciences
University of Oxford
Level 3, John Radcliffe Hospital,
Headley Way,
Oxford,
OX3 9DU
Email: james.malycha@ndcn.ox.ac.uk
Phone: work (+44) 1865 223 058 mobile 07983 093 758

Dr Oliver Redfern MBBS PhD
Post-Doctoral Research Fellow
Kadoorie Centre for Critical Care Research and Education
Nuffield Department of Clinical Neurosciences
University of Oxford
Level 3, John Radcliffe Hospital,
Headley Way,
Oxford,
OX3 9DU
Email: oliver.redfern@ndcn.ox.ac.uk

Professor Guy Ludbrook PhD MBBS FANZCA
Professor of Anaesthesia
University of Adelaide
Faculty of Health and Medical Science
North Terrace
AHMS Floor 8
5000
Email: guy.ludbrook@adelaide.edu.au

Professor J. Duncan Young DM FRCA
Professor of Intensive Care Medicine
Kadoorie Centre for Critical Care Research and Education

For peer review only - http://bmjopen.bmj.com/site/about/guidelines.xhtml

Nuffield Department of Clinical Neurosciences
University of Oxford
Level 3, John Radcliffe Hospital,
Headley Way,
Oxford,
OX3 9DU
Email: duncan.young@ndcn.ox.ac.uk

Professor Peter J. Watkinson MD ChB MRCP FDICM
Associate Professor of Intensive Care Medicine
Kadoorie Centre for Critical Care Research and Education
Nuffield Department of Clinical Neurosciences
University of Oxford
Level 3, John Radcliffe Hospital,
Headley Way,
Oxford,
OX3 9DU
Email: peter.watkinson@ndcn.ox.ac.uk

Word count
1690 words

ABSTRACT

Introduction
Traditional early warning scores (EWS) use vital sign derangements to detect clinical deterioration in patients treated on hospital wards. Combining vital signs with demographics and laboratory results improves EWS performance. We have developed the Hospital Alerting Via Electronic Noticeboard (HAVEN) system. HAVEN uses vital signs, as well as demographic, comorbidity and laboratory data from the electronic patient record (EPR), to quantify and rank the risk of unplanned admission to an Intensive Care Unit (ICU) within 24 hours for all ward patients.

The primary aim of this study is to find additional variables, potentially missed during development, which may improve HAVEN performance. These variables will be sought in the medical record of patients misclassified by the HAVEN algorithm during testing.

Methods
This will be a prospective, observational, cohort study conducted at the John Radcliffe Hospital, part of the Oxford University Hospitals National Health Service Foundation Trust in the United Kingdom.

Each day during the study periods, we will document all highly ranked patients (i.e. those with the highest risk for unplanned ICU admission) identified by the HAVEN system. After 48 hours we will review the progress of the identified patients. Patients who were subsequently *admitted* to the ICU will be removed from the study (as they will have been correctly classified by HAVEN). Highly ranked patients *not admitted* to ICU will undergo a structured medical notes review. Additionally, at the end of the study periods, all patients who had an unplanned ICU admission but whom HAVEN *failed to rank highly* will have a structured medical notes review.

For peer review only - http://bmjopen.bmj.com/site/about/guidelines.xhtml

The review will identify candidate variables, likely associated with unplanned ICU admission, not included in the HAVEN algorithm

Ethics and dissemination

Approval has been granted for gathering the data used in this study (South Central – Oxford C, Research Ethics Committee) REC reference: 16/SC/0264, 13th June 2016) and Confidentiality Advisory Group (16/CAG/0066).

Discussion

Our study will use a clinical expert conducting a structured medical notes review to identify variables, associated with unplanned ICU admission, not included in the development of the HAVEN algorithm. These variables will then be added to the algorithm and evaluated for potential performance gain. To our knowledge, this is the first study of this type. We anticipate that documenting the HAVEN development methods will assist other research groups developing similar technology.

Study registration

This is a sub-study of the primary project 'HAVEN' which was registered in the ISRCTN registry (ISRCTN12518261) on 27th July 2016.

KEYWORDS

Clinical deterioration, intensive care unit, critical care unit, predictive score, electronic patient record, qualitative medical note review

Strengths and limitations of the study

- The study methodology is in accordance with the STROBE guidelines
- We describe a method that combines algorithm testing with a structured medical notes review conducted by a clinical expert for the iterative improvement of a digital system that quantifies risk for unplanned Intensive Care Unit admission in all ward patients
- To our knowledge, this is the first study of this type

BACKGROUND

Introduction

Early Warning Score (EWS) systems, such as the National Early Warning Score (NEWS), combine abnormalities in patient vital signs into an aggregate score. [1] This score triggers a clinical response when a threshold is exceeded. Despite wide-scale adoption of EWS systems, significant clinical patient deterioration on hospital wards still occurs. [1,2] Additionally, high numbers of false alerts lead to alert 'fatigue' and inefficient use of response teams. [3] Adding additional clinical information to such systems, such as laboratory results and co-morbidities, improves specificity. [4–12] However, identifying and adding new variables requires a systematic approach to avoid needless complexity. [13]

We have developed a system to predict (within 24 hours) the need for unplanned Intensive Care Unit (ICU) admission in ward patients. It is called Hospital Alerting Via Electronic Noticeboard (HAVEN). [14] To identify potential variables for inclusion in HAVEN, we used a modified Delphi process and a systematic literature review. [15] Those identified variables that were

For peer review only - http://bmjopen.bmj.com/site/about/guidelines.xhtml

For peer review only

available within the EPR were extracted from datasets comprising all patients admitted to two National Health Service (NHS) Trusts. [12] We then used a machine learning method [16] to select the optimal combination of variables for the HAVEN algorithm. In contrast to EWS systems, HAVEN was not designed to produce alerts. Instead, HAVEN provides a list of patients in the hospital, ranked from most to least at risk of requiring an ICU admission. The intent is that HAVEN will improve patient safety by informing the use of clinical response teams.

Aims and Objectives

The primary aim of this study is to discover additional candidate variables, not recognised during the data driven derivation process, that would improve the performance of the HAVEN algorithm. We will review the medical records of *misclassified* patients, i.e. patients ranked highly by HAVEN but who were not admitted to the ICU; or patients who were never ranked highly by HAVEN but had an unplanned ICU admission.

The HAVEN risk score

The HAVEN risk score is calculated using both *static* and *dynamic* variables extracted in real-time from the EPR.

Static variables refer to patient-level data available at admission: age, gender, co-morbidities (classified according to the Elixhauser Co-morbidity index [17]), and Hospital Frailty Risk Score. [18] As diagnostic coding in the UK occurs after a patient has been discharged, the co-morbidity index and frailty scores are calculated using a patient's admissions over the previous two years.

Dynamic variables refer to measurements taken repeatedly during hospital admission, i.e. laboratory results and vital signs. The HAVEN risk score is currently updated according to the most recent measurements of: albumin, bilirubin, C-reactive protein (CRP), haemoglobin, platelets, white cell count, potassium, sodium, urea, creatinine, heart rate, systolic blood pressure, respiratory rate, body temperature, a neurological status assessment using either the Alert-Verbal-Painful-Unresponsive (AVPU) scale or the Glasgow Coma Scale (GCS), peripheral oxygen saturation from pulse oximetry (SpO_2) and the estimated fraction of inspired oxygen. [19] A patient's HAVEN score is re-calculated each time a new dynamic variable is received by the system and the score is further adjusted for the time since hospital admission.

METHODS

The study will be reported according to the Strengthening Reporting of Observational Studies in Epidemiology (STROBE) guidelines. [20]

Design and setting

This is a prospective, observational, cohort study conducted in the John Radcliffe Hospital, part of Oxford University Hospitals National Health Service Foundation Trust in the UK. The John Radcliffe Hospital is a tertiary hospital with over 800 beds and serves a population over 650,000 people, who are generally more affluent and with higher life expectancy than the national average. [21]

Data Collection

For peer review only - http://bmjopen.bmj.com/site/about/guidelines.xhtml

Data collection will occur during four, full, non-consecutive weeks in 2019. The notes review will be undertaken by a senior critical care physician. Patients who are discharged or die during the study period will have these details recorded. They will remain in the analysis dataset.

Participants

Eligibility criteria

All adult patients (16 years or over) admitted to any medical or surgical ward will be eligible for inclusion. We will exclude patients for whom a score cannot be generated (i.e. those with no recorded vital sign or laboratory measurements) and patients in groups not included in the datasets used for score development (i.e. obstetric and emergency department patients).

Sample size

We will sample two sub-groups of patients:

1. False High Rank (FHR)
2. False Low Rank (FLR)

The False High Rank (FHR) group will consist of patients ranked highly by HAVEN but who were not admitted to the ICU. To identify this group, we will record the five highest ranked patients on the HAVEN system at 9am each morning of the study. After 48 hours, we will remove any patients who were subsequently admitted to the ICU. The remaining patients' records will be reviewed.

The False Low Rank (FLR) group will be identified at the end of the study and consist of all patients who had an unplanned ICU admission during the study period and were not present in any of the daily high-ranking groups. These patients' records will also undergo a medical notes review.

The study will run for four non-consecutive weeks with an expected recruitment of between 130 and 150 patients.

Structured medical notes review

We will carry out a structured review of patient medical notes (electronic and paper-based) for the two sample groups described above. From these, we will construct a medical summary, looking specifically at patient-centred and system-based variables associated with decisions around ICU admission. We will use a modified version of the Hogan el al. qualitative note review techniques. [22] We will then conduct a thematic analysis of the extracted data. [23] It is expected that from within the themes the additional variables will be identified. Along with the *as yet unknown* variables, the following data will be extracted:

1. Primary diagnosis
2. Comorbidities and past medical history (where not available from previous admissions)
3. Any treatment limitations put in place and the reasons for these including "Do not attempt resuscitation" (DNAR) documents
4. Current medication
5. Radiological imaging
6. Point-of-care blood gas analysis
7. Clinical Frailty Score. [24]

For peer review only - http://bmjopen.bmj.com/site/about/guidelines.xhtml

Qualitative methods

Qualitative data (e.g. information in free text) will be analysed thematically, using methods of constant comparison. [25] A coding framework will be constructed to assist understanding of the data. We will use Nvivo Software (QSR International Pty Ltd, **www.qsrinternational.com**) to support the qualitative analysis process.

Patient safety and public involvement

As an observational study of patient records with no intervention, adverse events related to research interventions are not possible. In the event that inadequate care is identified during the structured medical note review, local NHS Trust protocols will be followed. Reviewers will act in accordance with the General Medical Councils Good Medical Practice Guidelines (2013). This action includes acting immediately if a patient is not receiving basic care to meet their needs. If patients are at risk because of inadequate premises, equipment or other resources, policies or systems, we will correct the matter if possible and raise our concerns in line with workplace policy. All measures will be documented as per local policies.

The HAVEN project has had two lay members on the management committee throughout. They have been involved in regular discussions regarding the aims and remit of the HAVEN project.

DISCUSSION

Main findings

This study will use structured medical notes review on ward patients misclassified by HAVEN to identify variables that, if added to the algorithm, may enhance performance.

Strengths and limitations of the study

This study is part of a project wide process to document HAVEN's development methodology such that it is thorough, transparent, repeatable, reportable and useful for other groups developing similar technology.

Unplanned ICU admission is an outcome measure subject to bias, such as the decision-making of individual physicians, local practice guidelines and bed availability. [26,27] This study is limited to one hospital and results may not be generalisable to other hospitals without external validation. Identified variables may not be available in the EPR and therefore not available for evaluation in the HAVEN algorithm.

Implications

To our knowledge, this is the first protocol to describe a study of this type. We hope this protocol it will assist future development of similar systems.

DECLARATIONS

Acknowledgements

JM would like to acknowledge the University of Adelaide, Department of Acute Medicine, who are administering his PhD.

Funding

For peer review only - http://bmjopen.bmj.com/site/about/guidelines.xhtml

This publication presents independent research supported by the Health Innovation Challenge Fund (HICF-R9-524; WT-103703/Z/14/Z), a parallel funding partnership between the Department of Health and Wellcome Trust. The views expressed in this publication are those of the author(s) and not necessarily those of the Department of Health or Wellcome Trust.

Ethics approval and consent

Health Research Authority (South Central – Oxford C, Research Ethics Committee [REC]) approval was obtained for gathering the data used in this study from the (REC reference: 16/SC/0264, 13th June 2016) and Confidentiality Advisory Group (16/CAG/0066). Informed consent will not be obtained from the patients, however patients who have requested that their data are not used for research purposes will be identified and removed from the study database. Patients will be allocated a study ID and all data transferred to the research database will have direct identifiable information removed. All documents will be stored securely and only accessible by study staff and authorised personnel. The study will comply with the UK Data Protection Act 2018.

Consent for publication

Not applicable.

Availability of data and materials

The datasets generated and analysed during the study will not be publicly available but anonymised extracts may be available from the corresponding author on reasonable request.

Competing Interests

PW is Chief Medical Officer for Sensyne Health (https://www.sensynehealth.com/). The remaining authors declare that they have no competing interests.

Publication policy

The Investigators will be involved in reviewing drafts of the manuscripts, abstracts, press releases and any other publications arising from the study. Authors will acknowledge that the study was funded by the Wellcome Trust/Department of Health. Authorship will be determined in accordance with the ICMJE guidelines and other contributors will be acknowledged.

Author contributions

JM and OR designed the study, undertook the methodological planning and wrote the protocol. DY and PW assisted in study design and GL commented on successive drafts of the manuscript. All authors read and approved the final manuscript.

REFERENCES

1 Royal College of Physicians (London). National Early Warning Score (NEWS) 2: Standardising the assessment of acute-illness severity in the NHS. 2017.

2 Care Quality Commission. Opening the door to change NHS safety culture and the need for transformation. 2018.

3 Curry J, Jungquist CR. A critical assessment of monitoring practices, patient deterioration, and alarm fatigue on inpatient wards: a review. *Patient Saf Surg*

For peer review only - http://bmjopen.bmj.com/site/about/guidelines.xhtml

2014;**8**:29. doi:10.1186/1754-9493-8-29

4 Churpek MM, Yuen TC, Park SY, *et al.* Using Electronic Health Record Data to Develop and Validate a Prediction Model for Adverse Outcomes in the Wards. *Crit Care Med* 2014;**42**:841–8. doi:10.1097/CCM.0000000000000038

5 Kang MA, Churpek MM, Zadravecz FJ, *et al.* Real-time risk prediction on the wards: A feasibility study. *Crit Care Med* 2016;**44**:1468–73. doi:10.1097/CCM.0000000000001716

6 Alvarez CA, Clark CA, Zhang S, *et al.* Predicting out of intensive care unit cardiopulmonary arrest or death using electronic medical record data. *BMC Med Inform Decis Mak* 2013;**13**:28.

7 Bailey TCC, Chen Y, Mao Y, *et al.* A trial of a real-time Alert for clinical deterioration in Patients hospitalized on general medical wards. *J Hosp Med* 2013;**8**:236–42. doi:10.1002/jhm.2009

8 Escobar GJ, LaGuardia JC, Turk BJ, *et al.* Early detection of impending physiologic deterioration among patients who are not in intensive care: Development of predictive models using data from an automated electronic medical record. *J Hosp Med* 2012;**7**:388–95. doi:10.1002/jhm.1929

9 Hackmann G, Chen M, Chipara O, *et al.* Toward a two-tier clinical warning system for hospitalized patients. *AMIA . Annu Symp proceedings AMIA Symp* 2011;**2011**:511–9. doi:10.7936/K70V8B1G

10 Tam V, Frost SA, Hillman KM, *et al.* Using administrative data to develop a nomogram for individualising risk of unplanned admission to intensive care. *Resuscitation* 2008;**79**:241–8. doi:10.1016/j.resuscitation.2008.06.023

11 Kipnis P, Turk BJ, Wulf DA, *et al.* Development and validation of an electronic medical record-based alert score for detection of inpatient deterioration outside the ICU. *J Biomed Inform* 2016;**64**:10–9. doi:10.1016/j.jbi.2016.09.013

12 Redfern OC, Pimentel MAF, Prytherch D, *et al.* Predicting in-hospital mortality and unanticipated admissions to the intensive care unit using routinely collected blood tests and vital signs: Development and validation of a multivariable model. *Resuscitation* 2018;**133**:75–81. doi:10.1016/j.resuscitation.2018.09.021

13 Collins GS, Reitsma JB, Altman DG, *et al.* Transparent reporting of a multivariable prediction model for individual prognosis or diagnosis (TRIPOD): The TRIPOD Statement. *Eur Urol* 2015;**67**:1142–51. doi:10.1016/j.eururo.2014.11.025

14 Watkinson, P., Young, J. D., Prytherch, D., Tarassenko, L., Clifton, D., & Briggs J. Hospital Alerting Via Electronic Noticeboard (HAVEN) Study Protocol. 2016.https://ora.ox.ac.uk/objects/uuid:322561c7-866e-4c

15 Malycha J, Bonnici T, Clifton DA, *et al.* Patient centred variables with univariate associations with unplanned ICU admission: a systematic review. *BMC Med Inform*

For peer review only - http://bmjopen.bmj.com/site/about/guidelines.xhtml

Decis Mak 2019;**19**:98. doi:10.1186/s12911-019-0820-1

16 Chen T, He T, Benesty M, *et al.* Extreme Gradient Boosting Package 'xgboost'. 2018;:54.

17 Quan H, Sundararajan V, Halfon P, *et al.* Coding algorithms for defining comorbidities in ICD-9-CM and ICD-10 administrative data. *Med Care* 2005;**43**:1130–9. doi:10.1097/01.mlr.0000182534.19832.83

18 Gilbert T, Neuburger J, Kraindler J, *et al.* Development and validation of a Hospital Frailty Risk Score focusing on older people in acute care settings using electronic hospital records: an observational study. *Lancet* 2018;**391**:1775–82. doi:10.1016/S0140-6736(18)30668-8

19 Malycha J, Farajidavar N, Pimentel MAF, *et al.* The effect of fractional inspired oxygen concentration on early warning score performance: A database analysis. *Resuscitation* 2019;**139**:192–9. doi:10.1016/j.resuscitation.2019.04.002

20 von Elm E, Altman DG, Egger M, *et al.* The Strengthening the Reporting of Observational Studies in Epidemiology (STROBE) statement: guidelines for reporting observational studies. *J Clin Epidemiol* 2008;**61**:344–9. doi:10.1016/j.jclinepi.2007.11.008

21 Public Health England. Oxford Health Profile. 2017.

22 Hogan H, Healey F, Neale G, *et al.* Preventable deaths due to problems in care in English acute hospitals: A retrospective case record review study. *BMJ Qual Saf* 2012;**21**:737–45. doi:10.1136/bmjqs-2011-001159

23 Braun, Virginia; Clarke V. Using thematic analysis in Psychology. *Qual Res Psychol* 2006;**3**:77–101. doi:10.1017/CBO9781107415324.004

24 Ke LS. Frailty in the elderly: A concept analysis. *J Nurs* 2013;**60**:105–10. doi:10.1503/cmaj.050051

25 Huo X, Liu C, Bai X, *et al.* RSC Advances Aqueous extract of Cordyceps sinensis potentiates the antitumor e ff ect of DDP and attenuates. *RSC Adv* 2017;**7**:37743–54. doi:10.1136/bmj.320.7227.114

26 Robert R, Coudroy R, Ragot S, *et al.* Influence of ICU-bed availability on ICU admission decisions. *Ann Intensive Care* 2015;**5**:1–7. doi:10.1186/s13613-015-0099-z

27 Robert R, Reignier J, Tournoux-Facon C, *et al.* Refusal of intensive care unit admission due to a full unit: Impact on mortality. *Am J Respir Crit Care Med* 2012;**185**:1081–7. doi:10.1164/rccm.201104-0729OC

For peer review only - http://bmjopen.bmj.com/site/about/guidelines.xhtml

TITLE PAGE
Authors
Affiliations
Word count

ABSTRACT
Introduction
Methods
Ethics and dissemination
Discussion
Study registration

KEYWORDS

STRENGTHS AND LIMITATIONS OF THE STUDY

BACKGROUND
Introduction
Aims and Objectives

METHODS
Design and setting
Data Collection
Participants
Structured medical notes review
Qualitative methods
Patient safety and public involvement

DISCUSSION
Main findings
Strengths and limitations of the study
Implications

DECLARATIONS
Acknowledgements
Funding
Ethics approval and consent
Consent for publication
Availability of data and materials
Competing Interests
Publication policy

For peer review only

For peer review only - http://bmjopen.bmj.com/site/about/guidelines.xhtml

Author contributions

REFERENCES

For peer review only

For peer review only - http://bmjopen.bmj.com/site/about/guidelines.xhtml

BMJ Open

Testing a digital system that ranks the risk of unplanned Intensive Care Unit admission in all ward patients: protocol for a prospective observational cohort study

Journal:	*BMJ Open*
Manuscript ID	bmjopen-2019-032429.R1
Article Type:	Protocol
Date Submitted by the Author:	07-Aug-2019
Complete List of Authors:	Malycha, James; University of Oxford, Nuffield Department of Clinical Neurosciences;<br>Redfern, Oliver; Oxford University, Nuffield Department of Clinical Neurosciences;<br>Ludbrook, Guy; University of Adelaide,<br>Young, Duncan; Oxford University, Nuffield Department of Clinical Neurosciences<br>Watkinson, Peter; Oxford University Hospitals NHS Trust, Kadoorie Centre for Critical Care research and Education
**Primary Subject Heading**:	Intensive care
Secondary Subject Heading:	Health informatics
Keywords:	Clinical deterioration, INTENSIVE & CRITICAL CARE, Predictive score, Electronic Patient Record

SCHOLARONE™
Manuscripts

TITLE PAGE

Testing a digital system that ranks the risk of unplanned Intensive Care Unit admission in all ward patients: protocol for a prospective observational cohort study

AUTHORS

Dr James Malycha (corresponding author) MBBS FCICM
Clinical Research Fellow
Kadoorie Centre for Critical Care Research and Education
Nuffield Department of Clinical Neurosciences
University of Oxford
Level 3, John Radcliffe Hospital,
Headley Way,
Oxford,
OX3 9DU

Postal address: Kadoorie Centre for Critical Care Research and Education
Nuffield Department of Clinical Neurosciences
University of Oxford
Level 3, John Radcliffe Hospital,
Headley Way,
Oxford,
OX3 9DU
Email: james.malycha@ndcn.ox.ac.uk
Phone: work (+44) 1865 223 058 mobile 07983 093 758

Dr Oliver Redfern MBBS PhD
Post-Doctoral Research Fellow
Kadoorie Centre for Critical Care Research and Education
Nuffield Department of Clinical Neurosciences
University of Oxford
Level 3, John Radcliffe Hospital,
Headley Way,
Oxford,
OX3 9DU
Email: oliver.redfern@ndcn.ox.ac.uk

Professor Guy Ludbrook PhD MBBS FANZCA
Professor of Anaesthesia
University of Adelaide
Faculty of Health and Medical Science
North Terrace
AHMS Floor 8
5000
Email: guy.ludbrook@adelaide.edu.au

Professor J. Duncan Young DM FRCA
Professor of Intensive Care Medicine
Kadoorie Centre for Critical Care Research and Education

For peer review only

For peer review only - http://bmjopen.bmj.com/site/about/guidelines.xhtml

Nuffield Department of Clinical Neurosciences
University of Oxford
Level 3, John Radcliffe Hospital,
Headley Way,
Oxford,
OX3 9DU
Email: duncan.young@ndcn.ox.ac.uk

Professor Peter J. Watkinson MD ChB MRCP FDICM
Associate Professor of Intensive Care Medicine
Kadoorie Centre for Critical Care Research and Education
Nuffield Department of Clinical Neurosciences
University of Oxford
Level 3, John Radcliffe Hospital,
Headley Way,
Oxford,
OX3 9DU
Email: peter.watkinson@ndcn.ox.ac.uk

Word count

1690 words

ABSTRACT

Introduction

Traditional early warning scores (EWS) use vital sign derangements to detect clinical deterioration in patients treated on hospital wards. Combining vital signs with demographics and laboratory results improves EWS performance. We have developed the Hospital Alerting Via Electronic Noticeboard (HAVEN) system. HAVEN uses vital signs, as well as demographic, comorbidity and laboratory data from the electronic patient record (EPR), to quantify and rank the risk of unplanned admission to an Intensive Care Unit (ICU) within 24 hours for all ward patients.

The primary aim of this study is to find additional variables, potentially missed during development, which may improve HAVEN performance. These variables will be sought in the medical record of patients misclassified by the HAVEN risk score during testing.

Methods

This will be a prospective, observational, cohort study conducted at the John Radcliffe Hospital, part of the Oxford University Hospitals National Health Service Foundation Trust in the United Kingdom.

Each day during the study periods, we will document all highly ranked patients (i.e. those with the highest risk for unplanned ICU admission) identified by the HAVEN system. After 48 hours we will review the progress of the identified patients. Patients who were subsequently *admitted* to the ICU will be removed from the study (as they will have been correctly classified by HAVEN). Highly ranked patients *not admitted* to ICU will undergo a structured medical notes review. Additionally, at the end of the study periods, all patients who had an unplanned ICU admission but whom HAVEN *failed to rank highly* will have a structured medical notes review.

For peer review only - http://bmjopen.bmj.com/site/about/guidelines.xhtml

The review will identify candidate variables, likely associated with unplanned ICU admission, not included in the HAVEN risk score

Ethics and dissemination

Approval has been granted for gathering the data used in this study (South Central – Oxford C, Research Ethics Committee) REC reference: 16/SC/0264, 13th June 2016) and Confidentiality Advisory Group (16/CAG/0066).

Discussion

Our study will use a clinical expert conducting a structured medical notes review to identify variables, associated with unplanned ICU admission, not included in the development of the HAVEN risk score. These variables will then be added to the risk score and evaluated for potential performance gain. To our knowledge, this is the first study of this type. We anticipate that documenting the HAVEN development methods will assist other research groups developing similar technology.

Study registration

This is a sub-study of the primary project 'HAVEN' which was registered in the ISRCTN registry (ISRCTN12518261) on 27th July 2016.

KEYWORDS

Clinical deterioration, intensive care unit, critical care unit, predictive score, electronic patient record, qualitative medical note review

Strengths and limitations of the study

- The study methodology is in accordance with the STROBE guidelines
- We describe a method that combines risk score testing with a structured medical notes review conducted by a clinical expert for the iterative improvement of a digital system that quantifies risk for unplanned Intensive Care Unit admission in all ward patients
- To our knowledge, this is the first study of this type

BACKGROUND

Introduction

Early Warning Score (EWS) systems, such as the National Early Warning Score (NEWS), combine abnormalities in patient vital signs into an aggregate score. [1] This score triggers a clinical response when a threshold is exceeded. Despite wide-scale adoption of EWS systems, significant clinical patient deterioration on hospital wards still occurs. [1,2] Additionally, high numbers of false alerts lead to alert 'fatigue' and inefficient use of response teams. [3] Adding additional clinical information to such systems, such as laboratory results and co-morbidities, improves specificity. [4–12] However, identifying and adding new variables requires a systematic approach to avoid needless complexity. [13]

We have developed a system to predict the risk of unplanned ICU admission (within 24 hours) for patients on general medical and surgical wards. It is called Hospital Alerting Via Electronic Noticeboard (HAVEN). [14] To identify potential variables for inclusion in HAVEN, we used a modified Delphi process and a systematic literature review. [15] Those identified variables

For peer review only - http://bmjopen.bmj.com/site/about/guidelines.xhtml

that were available within the Electronic Patient Record (EPR) were extracted from datasets comprising all patients admitted to two National Health Service (NHS) Trusts (a Trust is a legal entity that provides goods and services for the purposes of the provision of hospital, community and/or other aspects of patient care). [12] We then used a machine learning method [16] to select the optimal combination of variables for the HAVEN risk score. In contrast to EWS systems, HAVEN was not designed to produce alerts. Instead, HAVEN provides a list of patients in the hospital, ranked from most to least at risk of requiring an ICU admission. The intent is that HAVEN will improve patient safety by informing the use of clinical response teams.

Aims and Objectives

The primary aim of this study is to discover additional candidate variables, not recognised during the data driven derivation process that would improve the performance of the HAVEN risk score. We will review the medical records of *misclassified* patients, i.e. patients ranked highly by HAVEN but who were not admitted to the ICU; or patients who were never ranked highly by HAVEN but had an unplanned ICU admission.

The HAVEN risk score

The HAVEN risk score is calculated using both *static* and *dynamic* variables extracted in real-time from the EPR.

Static variables refer to patient-level data available at admission: age, gender, co-morbidities (classified according to the Elixhauser Co-morbidity index [17]), and Hospital Frailty Risk Score. [18] As diagnostic coding in the United Kingdom (UK) occurs after a patient has been discharged, the co-morbidity index and frailty scores are calculated using a patient's admissions over the previous two years. Score performance in patients with no previous admissions (and potentially undocumented comorbidities) will be evaluated separately.

Dynamic variables refer to measurements taken repeatedly during hospital admission, i.e. laboratory results and vital signs. The HAVEN risk score is currently updated according to the most recent measurements of: albumin, bilirubin, C-reactive protein (CRP), haemoglobin, platelets, white cell count, potassium, sodium, urea, creatinine, heart rate, systolic blood pressure, respiratory rate, body temperature, a neurological status assessment using either the Alert-Verbal-Painful-Unresponsive (AVPU) scale or the Glasgow Coma Scale (GCS), peripheral oxygen saturation from pulse oximetry (SpO_2) and the estimated fraction of inspired oxygen. [19] A patient's HAVEN score is re-calculated each time a new dynamic variable is received by the system and the score is further adjusted for the time since hospital admission.

METHODS

The study will be reported according to the Strengthening Reporting of Observational Studies in Epidemiology (STROBE) guidelines. [20]

Design and setting

This is a prospective, observational, cohort study conducted in the John Radcliffe Hospital, part of Oxford University Hospitals National Health Service Foundation Trust in the UK. The John Radcliffe Hospital is a tertiary hospital with over 800 beds and serves a population over 650,000 people, who are generally more affluent and with higher life expectancy than the national average. [21]

For peer review only - http://bmjopen.bmj.com/site/about/guidelines.xhtml

Data Collection

Data collection will occur during four, full, non-consecutive weeks in 2019. The notes review will be undertaken by a senior critical care physician. Patients who are discharged or die during the study period will have these details recorded. They will remain in the analysis dataset.

Participants

Eligibility criteria

Emergency and elective adult patients (16 years or over) admitted to medical, surgical, observational or short stay wards will be eligible for inclusion. We will exclude patients for whom a score cannot be generated (i.e. those with no recorded vital sign or laboratory measurements).

Sample size

We will sample two sub-groups of patients:

1. False High Rank (FHR)
2. False Low Rank (FLR)

The False High Rank (FHR) group will consist of patients ranked highly by HAVEN but who were not admitted to the ICU. To identify this group, we will record the five highest ranked patients on the HAVEN system at 9am each morning of the study. After 48 hours, we will remove any patients who were subsequently admitted to the ICU. The remaining patients' records will be reviewed.

The False Low Rank (FLR) group will be identified at the end of the study and consist of all patients who had an unplanned ICU admission during the study period and were not present in any of the daily high-ranking groups. These patients' records will also undergo a medical notes review.

The study will run for four non-consecutive weeks with an expected recruitment of between 130 and 150 patients.

Structured medical notes review

We will carry out a structured review of patient medical notes (electronic and paper-based) for the two sample groups described above. From these, we will construct a medical summary, looking specifically at patient-centred and system-based variables associated with decisions around ICU admission. We will use a modified version of the Hogan el al. qualitative note review techniques. [22] We will then conduct a thematic analysis of the extracted data. [23] It is expected that from within the themes the additional variables will be identified. Along with the *as yet unknown* variables, the following data will be extracted:

1. Primary diagnosis
2. Comorbidities and past medical history (where not available from previous admissions)
3. Any treatment limitations put in place and the reasons for these including "Do not attempt resuscitation" (DNAR) documents
4. Current medication
5. Radiological imaging
6. Point-of-care blood gas analysis
7. Clinical Frailty Score. [24]

For peer review only - http://bmjopen.bmj.com/site/about/guidelines.xhtml

Qualitative methods

Qualitative data (e.g. information in free text) will be analysed thematically, using methods of constant comparison. [25] A coding framework will be constructed to assist understanding of the data. We will use Nvivo Software (QSR International Pty Ltd, www.qsrinternational.com) to support the qualitative analysis process.

Patient safety and public involvement

As an observational study of patient records with no intervention, adverse events related to research interventions are not possible. In the event that inadequate care is identified during the structured medical note review, local NHS Trust protocols will be followed. Reviewers will act in accordance with the General Medical Councils Good Medical Practice Guidelines (2013). This action includes acting immediately if a patient is not receiving basic care to meet their needs. If patients are at risk because of inadequate premises, equipment or other resources, policies or systems, we will correct the matter if possible and raise our concerns in line with workplace policy. All measures will be documented as per local policies. The HAVEN project has had two lay members on the management committee throughout. They have been involved in regular discussions regarding the aims and remit of the HAVEN project.

DISCUSSION

Main findings

This study will use structured medical notes review on ward patients misclassified by HAVEN to identify variables that may enhance performance. Any identified variables will be systematically introduced into our score development pipeline to evaluate whether they improve score performance.

Strengths and limitations of the study

This study is part of a project-wide process to document the development of the HAVEN system such that it is thorough, transparent, repeatable, reportable and the methodology could be useful for other groups developing similar technology.

Unplanned ICU admission is an outcome measure subject to bias, such as the decision-making of individual physicians, local practice guidelines and bed availability. [26,27] This study is limited to one hospital and the results may not be generalisable to other hospitals. Variables identified from the thematic analysis may not be available in the EPR and therefore cannot risk score be used to improve the performance of the HAVEN risk score. Likewise, patients with no previous admissions to the John Radcliffe Hospital will have no available comorbidity data, potentially limiting performance of the risk score in these patients. To assess the impact of these missing data, we will undertake sub-groups analyses in those patients with/without prior admissions.

While a significant proportion of ICU admissions are referred directly from the Emergency Department (ED), the HAVEN system was designed specifically for ward patients needing the attention of the critical care team. By excluding these ED referrals we are reducing the number of eligible patients for this study.

Implications

For peer review only - http://bmjopen.bmj.com/site/about/guidelines.xhtml

To our knowledge, this is the first protocol to describe a study of this type. We hope this protocol it will assist future development of similar systems.

DECLARATIONS

Acknowledgements

JM would like to acknowledge the University of Adelaide, Department of Acute Medicine, who are administering his PhD.

Funding

This publication presents independent research supported by the Health Innovation Challenge Fund (HICF-R9-524; WT-103703/Z/14/Z), a parallel funding partnership between the Department of Health and Wellcome Trust. The views expressed in this publication are those of the author(s) and not necessarily those of the Department of Health or Wellcome Trust.

Ethics approval and consent

Health Research Authority (South Central – Oxford C, Research Ethics Committee [REC]) approval was obtained for gathering the data used in this study from the (REC reference: 16/SC/0264, 13th June 2016) and Confidentiality Advisory Group (16/CAG/0066). Informed consent will not be obtained from the patients, however patients who have requested that their data are not used for research purposes will be identified and removed from the study database. Patients will be allocated a study ID and all data transferred to the research database will have direct identifiable information removed. All documents will be stored securely and only accessible by study staff and authorised personnel. The study will comply with the UK Data Protection Act 2018.

Consent for publication

Not applicable.

Availability of data and materials

The datasets generated and analysed during the study will not be publicly available but anonymised extracts may be available from the corresponding author on reasonable request.

Competing Interests

PW is Chief Medical Officer for Sensyne Health (https://www.sensynehealth.com/). The remaining authors declare that they have no competing interests.

Publication policy

The Investigators will be involved in reviewing drafts of the manuscripts, abstracts, press releases and any other publications arising from the study. Authors will acknowledge that the study was funded by the Wellcome Trust/Department of Health. Authorship will be determined in accordance with the ICMJE guidelines and other contributors will be acknowledged.

Author contributions

For peer review only

JM and OR designed the study, undertook the methodological planning and wrote the protocol. DY and PW assisted in study design and GL commented on successive drafts of the manuscript. All authors read and approved the final manuscript.

REFERENCES

1 Royal College of Physicians (London). National Early Warning Score (NEWS) 2: Standardising the assessment of acute-illness severity in the NHS. 2017.

2 Care Quality Commission. Opening the door to change NHS safety culture and the need for transformation. 2018.

3 Curry J, Jungquist CR. A critical assessment of monitoring practices, patient deterioration, and alarm fatigue on inpatient wards: a review. *Patient Saf Surg* 2014;**8**:29. doi:10.1186/1754-9493-8-29

4 Churpek MM, Yuen TC, Park SY, *et al.* Using Electronic Health Record Data to Develop and Validate a Prediction Model for Adverse Outcomes in the Wards. *Crit Care Med* 2014;**42**:841–8. doi:10.1097/CCM.0000000000000038

5 Kang MA, Churpek MM, Zadravecz FJ, *et al.* Real-time risk prediction on the wards: A feasibility study. *Crit Care Med* 2016;**44**:1468–73. doi:10.1097/CCM.0000000000001716

6 Alvarez CA, Clark CA, Zhang S, *et al.* Predicting out of intensive care unit cardiopulmonary arrest or death using electronic medical record data. *BMC Med Inform Decis Mak* 2013;**13**:28.

7 Bailey TCC, Chen Y, Mao Y, *et al.* A trial of a real-time Alert for clinical deterioration in Patients hospitalized on general medical wards. *J Hosp Med* 2013;**8**:236–42. doi:10.1002/jhm.2009

8 Escobar GJ, LaGuardia JC, Turk BJ, *et al.* Early detection of impending physiologic deterioration among patients who are not in intensive care: Development of predictive models using data from an automated electronic medical record. *J Hosp Med* 2012;**7**:388–95. doi:10.1002/jhm.1929

9 Hackmann G, Chen M, Chipara O, *et al.* Toward a two-tier clinical warning system for hospitalized patients. *AMIA . Annu Symp proceedings AMIA Symp* 2011;**2011**:511–9. doi:10.7936/K70V8B1G

10 Tam V, Frost SA, Hillman KM, *et al.* Using administrative data to develop a nomogram for individualising risk of unplanned admission to intensive care. *Resuscitation* 2008;**79**:241–8. doi:10.1016/j.resuscitation.2008.06.023

11 Kipnis P, Turk BJ, Wulf DA, *et al.* Development and validation of an electronic medical record-based alert score for detection of inpatient deterioration outside the ICU. *J Biomed Inform* 2016;**64**:10–9. doi:10.1016/j.jbi.2016.09.013

12 Redfern OC, Pimentel MAF, Prytherch D, *et al.* Predicting in-hospital mortality and

For peer review only

For peer review only - http://bmjopen.bmj.com/site/about/guidelines.xhtml

unanticipated admissions to the intensive care unit using routinely collected blood tests and vital signs: Development and validation of a multivariable model. *Resuscitation* 2018;**133**:75–81. doi:10.1016/j.resuscitation.2018.09.021

13 Collins GS, Reitsma JB, Altman DG, *et al.* Transparent reporting of a multivariable prediction model for individual prognosis or diagnosis (TRIPOD): The TRIPOD Statement. *Eur Urol* 2015;**67**:1142–51. doi:10.1016/j.eururo.2014.11.025

14 Watkinson, P., Young, J. D., Prytherch, D., Tarassenko, L., Clifton, D., & Briggs J. Hospital Alerting Via Electronic Noticeboard (HAVEN) Study Protocol. 2016.https://ora.ox.ac.uk/objects/uuid:322561c7-866e-4c

15 Malycha J, Bonnici T, Clifton DA, *et al.* Patient centred variables with univariate associations with unplanned ICU admission: a systematic review. *BMC Med Inform Decis Mak* 2019;**19**:98. doi:10.1186/s12911-019-0820-1

16 Chen T, He T, Benesty M, *et al.* Extreme Gradient Boosting Package 'xgboost'. 2018;:54.

17 Quan H, Sundararajan V, Halfon P, *et al.* Coding algorithms for defining comorbidities in ICD-9-CM and ICD-10 administrative data. *Med Care* 2005;**43**:1130–9. doi:10.1097/01.mlr.0000182534.19832.83

18 Gilbert T, Neuburger J, Kraindler J, *et al.* Development and validation of a Hospital Frailty Risk Score focusing on older people in acute care settings using electronic hospital records: an observational study. *Lancet* 2018;**391**:1775–82. doi:10.1016/S0140-6736(18)30668-8

19 Malycha J, Farajidavar N, Pimentel MAF, *et al.* The effect of fractional inspired oxygen concentration on early warning score performance: A database analysis. *Resuscitation* 2019;**139**:192–9. doi:10.1016/j.resuscitation.2019.04.002

20 von Elm E, Altman DG, Egger M, *et al.* The Strengthening the Reporting of Observational Studies in Epidemiology (STROBE) statement: guidelines for reporting observational studies. *J Clin Epidemiol* 2008;**61**:344–9. doi:10.1016/j.jclinepi.2007.11.008

21 Public Health England. Oxford Health Profile. 2017.

22 Hogan H, Healey F, Neale G, *et al.* Preventable deaths due to problems in care in English acute hospitals: A retrospective case record review study. *BMJ Qual Saf* 2012;**21**:737–45. doi:10.1136/bmjqs-2011-001159

23 Braun, Virginia; Clarke V. Using thematic analysis in Psychology. *Qual Res Psychol* 2006;**3**:77–101. doi:10.1017/CBO9781107415324.004

24 Ke LS. Frailty in the elderly: A concept analysis. *J Nurs* 2013;**60**:105–10. doi:10.1503/cmaj.050051

25 Huo X, Liu C, Bai X, *et al.* RSC Advances Aqueous extract of Cordyceps sinensis

For peer review only

For peer review only - http://bmjopen.bmj.com/site/about/guidelines.xhtml

potentiates the antitumor e ff ect of DDP and attenuates. *RSC Adv* 2017;**7**:37743–54. doi:10.1136/bmj.320.7227.114

26 Robert R, Coudroy R, Ragot S, *et al.* Influence of ICU-bed availability on ICU admission decisions. *Ann Intensive Care* 2015;**5**:1–7. doi:10.1186/s13613-015-0099-z

27 Robert R, Reignier J, Tournoux-Facon C, *et al.* Refusal of intensive care unit admission due to a full unit: Impact on mortality. *Am J Respir Crit Care Med* 2012;**185**:1081–7. doi:10.1164/rccm.201104-0729OC

For peer review only

For peer review only - http://bmjopen.bmj.com/site/about/guidelines.xhtml

TITLE PAGE
Authors
Affiliations
Word count

ABSTRACT
Introduction
Methods
Ethics and dissemination
Discussion
Study registration

KEYWORDS

STRENGTHS AND LIMITATIONS OF THE STUDY

BACKGROUND
Introduction
Aims and Objectives

METHODS
Design and setting
Data Collection
Participants
Structured medical notes review
Qualitative methods
Patient safety and public involvement

DISCUSSION
Main findings
Strengths and limitations of the study
Implications

DECLARATIONS
Acknowledgements
Funding
Ethics approval and consent
Consent for publication
Availability of data and materials
Competing Interests
Publication policy

For peer review only - http://bmjopen.bmj.com/site/about/guidelines.xhtml

Author contributions

REFERENCES

For peer review only

For peer review only - http://bmjopen.bmj.com/site/about/guidelines.xhtml
